# The Treatment of Wounds

**Published:** 1877-10-20

**Authors:** Samson Gamgee

**Affiliations:** F. R. S., Edin., Surgeon to the Queen’s Hospital, Birmingham


### THE TREATMENT OF WOUNDS.

By SAMSON GAMGEE, ESQ., F. R. S., Edin., Surgeon to the Queen's Hospital, Birmingham.

[We generally learn most about principles of treatment from the simplest cases. A "little old gentleman" consulted Mr. Gamgee on account of a cystic tumor about the size of a hen's egg, in the right temporal region.]

The skin was very red, tense, and painful, and the hat, though a very soft one, was worn with much difficulty. After transfixing the growth vertically through the base, and peeling out the two halves of the cyst, with its bread-sauce-like contents, I dried the interior of the wound with a fine sponge. The edges were then very accurately approximated, and kept so with a few strips of lint soaked in styptic colloid. A few turns of bandage completed the dressing. When I removed it, at the end of five days, there was not a drop of discharge, adhesion was perfect, and afforded a simple but complete illustration of the surgeon's first intention in treating wounds—to secure direct union. All that is visible of the cicatrix is a very fine, pinkish line, extending upwards about two inches from the right ear.

Please to note—firstly, that the wound was thoroughly dried with a fine sponge; secondly, styptic colloid was used to keep the edges in contact; thirdly, the parts were not disturbed until the fifth day, when union was complete and solid.

Drenching wounds with water during an operation, and washing them with it afterwards, are mistakes. Water favors decomposition, which is the enemy of healing action.

The styptic colloid, used to keep the edges of the wounds together, is the admirable preparation introduced in 1867 by my friend Dr. B. W. Richardson. (Styptic colloid, as prepared after the instructions of Dr. Richardson, by Messrs. Robbiins & Co., of Oxford street, is produced by saturating ether entirely with tannin and a colloidal substance, xyloidine, or gun-cotton, a little tincture of benzoin being finally admixed.) In removing the styptic colloid dressing, common water should be scrupulously avoided, and a mixture of alcohol and ether employed, or equal parts of absolute alcohol and distilled water, warmed to a little above the heat of the body.

It has been noted that the dressing was not touched for five days after the operation. Once divided parts—be they hard or soft, bones or muscles, skin or nerves—are adjusted with a view to union, the less they are disturbed the better.

A case illustrating the same principles, though on a somewhat larger scale, is that of C. H., aged forty-three, who was lately in Ward 5, whose right breast I removed on the 20th of May, with a small hard gland from the corresponding axilla. Of the operation, it only need be said that, according to my usual practice, I cut down upon the sternal origin of the great pectoral and dissected it clean, so as to make sure of thorough removal of the dieased mass. I am convinced that many so-called rapid recurrences of cancer are only growths of pieces left behind, and that *thoroughness* is the very essence of success in extirpation of malignant growths. After removal of the breast, the edges of the wound were neatly brought together by numerous points of silver suture, and dressed with a layer of fine cotton wool, and over it picked oakum. An evenly compressing bandage was then applied around the chest, and made to include the arm and hand in the flexed position so as to fix them immovably to the side. The first night the temperature rose to 101.3°, but it never rose afterwards above 100°.

The wound was first dressed at the end of the fifth day after the operation. A great part of it being healed by the first intention, a large number of the sutures were removed, and strips of adhesive plaster applied so as to keep the edges in apposition; a pledget of oakum with a compressing bandage completed the dressing. On June 1st, the remaining sutures were removed. The wound was then nearly all healed, and the same dressing applied. On June 3d (fourteenth day after the operation), the entry on the card is, "Patient dressed (as before), and sent home well."

The points in this case to which I wish to direct your attention are—(*a*) the numerous sutures; (*b*) the cotton wool and picked oakum dressing; (*c*) the compressing bandage; (*d*) the rare dressing.

Metallic sutures so very rarely cause any irritation, that they may be inserted very near each other with impunity. Sutures far apart, with gaping intervals, are comparatively useless. If the cut surfaces are to adhere they must be brought into contact and kept there, and for this purpose metallic sutures, half an inch apart, or even less, are most efficacious. I often apply intervening strips of lint soaked in styptic colloid, but in this case only placed over the wound a layer of fine cotton wool, and a pledget of picked oakum. The best cotton wool for surgical dressings is that sold for jewelers in thin sheets, about eighteen inches by twelve, with alternate layers of tissue-paper. You will often see claims of priority for cotton wool dressing. I do not pretend to say who first introduced it, but the credit of generalizing its application in the treatment of a great variety of surgical injuries chiefly belongs to Burggraeve, of Ghent.

It has been recorded that the breast case was only dressed three times, in the fortnight which elapsed between the operation and the patient's discharge, in accordance with the principle of infrequent dressing, of the minimum of disturbance to insure the maximum of rest, dwelt upon in the preceding case, and equally borne out by those to be presently brought to your notice. To the same end the smoothly and lightly compressing bandage round the chest very powerfully contributed. Of all surgical agencies, none so beneficent as compression, none requiring more delicate manipulation, none so inadequately appreciated. Under a smooth and uniformly, while lightly-compressing bandage, extravasations of blood are absorbed, the healing action is promoted, and a soothing influence is exercised. There must be no constriction—only equable adaptation of surface to surface with the light pressure, which always comforts. There must be no squeezing like that of an old college friend's hand when seen after long absence; such pressure as that, if continued, is intolerable constriction. The soothing surgical pressure is like that which you interchange with the hand of a lady, the pleasure of whose meeting is tempered by the respectful regard which she inspires. Your hand adapts itself to hers, and gently presses it wherever it can touch it, but nowhere squeezes it for fear of offending. Such pressure as that, when employed by the surgeon in the treatment of injuries, always soothes and heals.

To apply a nicely compressing bandage well, you must practice hundreds and hundreds of times, bearing in mind that in surgical, as in all art, the greatest results are often obtainable from the simplest means, provided they be emplayed with the skill which can only result from the most patient assiduity.—*Lancet*, Dec. 23, 1876, p. 885.—*Braithwaite.*

